# Non-invasive load monitoring based on deep learning to identify unknown loads

**DOI:** 10.1371/journal.pone.0296979

**Published:** 2024-02-09

**Authors:** Anshun Su, Zehua Du, Bo Yin

**Affiliations:** College of Information Science and Engineering, Ocean University of China, Qingdao, China; Dr. Mahalingam College of Engineering and Technology, INDIA

## Abstract

With the rapid development of smart grids, society has become increasingly urgent to solve the problems of low energy utilization efficiency and high energy consumption. In this context, load identification has become a key element in formulating scientific and effective energy consumption plans and reducing unnecessary energy waste. However, traditional load identification methods mainly focus on known electrical equipment, and accurate identification of unknown electrical equipment still faces significant challenges. A new encoding feature space based on Triplet neural networks is proposed in this paper to detect unknown electrical appliances using convex hull coincidence degree. Additionally, transfer learning is introduced for the rapid updating of the pre-classification model’s self-incrementing class with the unknown load. In experiments, the effectiveness of our method is successfully tested on the PLAID dataset. The accuracy of unknown load identification reached 99.23%. Through this research, we expect to bring a new idea to the field of load identification to meet the urgent need for the identification of unknown electrical appliances in the development of smart grids.

## Introduction

Global electricity consumption is continuously increasing. The 2021 global electricity data report reveals a staggering cumulative worldwide electricity consumption of 28.466 trillion kWh [1], emphasizing the immense scale of global energy usage. The energy demand is escalating, highlighting the significance of efficient energy utilization. To optimize electric energy utilization, load monitoring is a vital technical approach. Research consistently indicates that when residents can monitor and flexibly allocate power resources, there is a significant reduction in energy losses and an improvement in the overall utilization rate of electric energy [2–4]. Load monitoring methods can be categorized into two types: invasive load monitoring and non-invasive load monitoring. Invasive load monitoring involves the installation of power consumption detection devices on individual electrical appliances. This method offers high accuracy in extracting detailed power information for each appliance [5, 6]. However, due to the high cost of installing a power consumption detection device for every single device, it is not suitable for widespread adoption and practical application. The concept of non-intrusive load monitoring (NILM) was initially introduced by Hart [7] in a published article in 1992. NILM effectively addresses the cost issue associated with equipment deployment. This method involves the installation of an electricity detection device near the meter, which collects data that is subsequently analyzed using algorithms. By making minor modifications to the existing circuit structure at a low cost. NILM eliminates the need for individual power consumption detection devices on each appliance. However, more advanced algorithms are needed for precise analysis of data. The choice between invasive and non-invasive load monitoring methods depends on specific application requirements and constraints. Invasive methods provide high-precision data and are suitable for applications with strict power consumption information requirements. However, they are costly and not suitable for large-scale deployment. Non-invasive methods have lower costs and broader applicability but may require more complexity in algorithms and data analysis. They may not offer the same level of accuracy as invasive methods. Therefore, when selecting a monitoring method, after weighing these factors, the ultimate choice is non-invasive detection.

In recent years, the application of deep learning methods in non-invasive load monitoring has shown significant growth. Deep learning methods can understand the deep structures within data, enhancing the performance of models. Additionally, deep learning models are highly flexible in capturing nonlinear relationships within data, which is particularly advantageous for tackling complex problems. Deep learning has become one of the primary applications in the field of load identification. In the majority of non-intrusive load identification algorithms, appliance identification typically occurs when the load category and the number of loads are already known. Guo L, Wang [8] et al. proposed a load identification method based on active deep learning and discrete wavelet transform. Arash [9] et al. proposed a method utilizing a convolutional neural network based on deep learning, employing a layered structure and feature extraction from power consumption curves to achieve appliance type detection and load disaggregation. Weicheng Liu [10] et al. proposed a time-domain power hybrid algorithm and a temporal convolutional autoencoder model to enhance the data processing accuracy of NILM. Eduardo [11] et al. applied the pinball quantile loss function to guide a deep neural network in NILM. Dong Ding [12] et al. proposed a method that utilizes multiple overlapping sliding windows and an improved convolutional neural network internal structure to effectively disaggregate highly mixed loads of multiple appliances. Xiao Zhou [13] et al. employed a deep learning model combining convolutional neural network, long short-term memory network, and random forest algorithms, effectively improving the accuracy of electrical appliance recognition. Leitao Qu [14] et al. proposed a residual convolutional neural network with energy normalization and squeeze-and-excitation blocks, applied in NILM.

But most load identification research currently concentrates on known electrical appliances with identified types and data characteristics. However, there is relatively less research dedicated to identifying unknown loads. Baets [15] proposed a clustering method based on the Siamese neural network for unknown device detection. The advantage of this method is that there is no mandatory limit on the number of equipment types, and there is no need to pre-set the number of clusters. M. Yu [16] et al. proposed a non-invasive load identification model based on the Siamese neural network. The model calculates the similarity of V-I trajectories using the Siamese network and dynamically incorporates new features into the feature library to identify unknown loads. Both methods utilize Siamese neural networks. However, training the Siamese neural network requires pairs of images to determine whether they belong to the same category or not. Moreover, the network may encounter challenges in distinguishing targets with high similarity. Bo Yin [17] et al. utilized the Siamese neural network for unknown device detection based on steady-state single-cycle current. In load identification, V-I trajectory provides more comprehensive information than single cycle current. For example, current waveform, phase information, frequency components, etc. Through these features, it is possible to describe the load behavior more accurately. Additionally, V-I trajectories are very useful for detecting and identifying non-linear loads. The V-I trajectory captures the nonlinear nature of the waveform. A single cycle current may not provide enough information. This method also uses the Siamese neural network and also has the disadvantages of the above two methods. Triplet neural network is typically more suitable for multi-class problems in load identification. It can reduce the labeling data cost, enhance generalization, and adapt to imbalanced data distributions. It effectively addresses the limitations of Siamese neural networks.

## Methods

### Feature extraction

The load characteristics can generally be divided into two types: steady-state characteristics [18] and transient characteristics [19] according to the different states of the load. In non-intrusive load identification, the characteristics of the load to be identified are determined by two factors: the electronic components present in the load equipment and its internal circuit structure. The steady-state characteristics and transient characteristics generally mentioned in power system research are more common in fault analysis and diagnosis [18, 20, 21]. Steady-state characteristics are typically more stable and less susceptible to noise and measurement errors, which can result in more reliable outcomes. Measurements of transient characteristics may be influenced by external disturbances. They may also require higher sampling frequencies. Additionally, more complex instrumentation might be needed. These factors have the potential to increase experimental uncertainties. Steady-state data is generally easier to process and analyze since it does not contain momentary fluctuations or noise. This facilitates researchers in extracting valuable information and trends from the data more easily. Different from the characteristics in system research, the non-intrusive load characteristics are more microscopic and correspond to a single load device. In this paper, steady-state features are selected for experiments.

**Fig 1:**
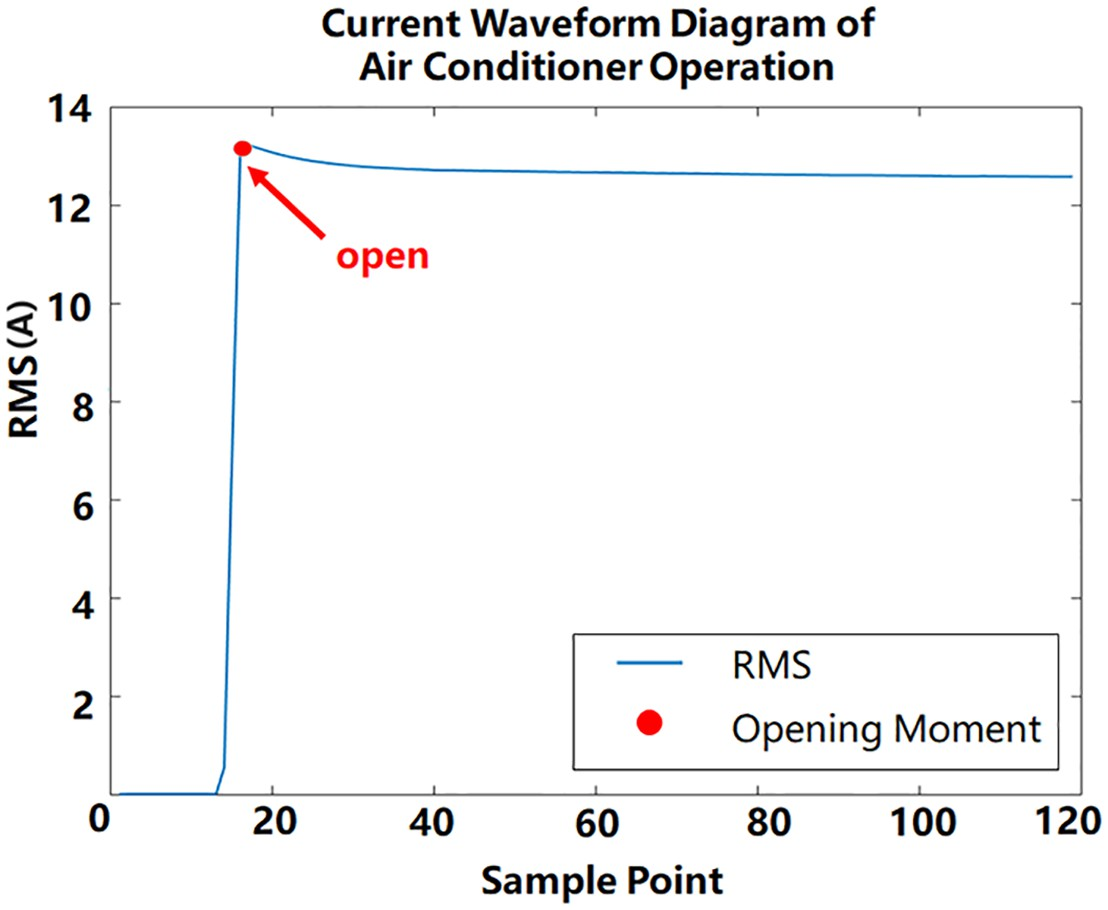
Running RMS current value of air conditioner.

Event detection is required before feature extraction. Understanding the events of electrical equipment under different operating states is crucial. Every time the electrical status changes, an event occurs. Fig 1 is an electrical operation waveform diagram drawn using the root mean square current(RMS) [22]. The red dot is the moment when the event occurs. Under the conditions of determining the turning on or off event of the electrical equipment, the interval range in the stable state is obtained. Anderson [23]et al. provide a framework for evaluating event detection algorithms in non-intrusive load monitoring. The accuracy of event detection is the basis for steady-state feature extraction. After obtaining the stable operation interval of the electrical appliance, we extract various steady-state characteristics of the electrical appliance.

Based on the stable state of operation, each electrical appliance extracts the current and voltage under different complete cycles and then normalizes the extracted data. The calculation formula is described in formula (1)

$$\begin{array}{c} \begin{array}{c} I_{m}\left(t\right)=\frac{I\left(t\right)-I_{min}}{I_{max}-I_{min}}\\ V_{m}\left(t\right)=\frac{V\left(t\right)-V_{min}}{V_{max}-V_{min}} \end{array} \end{array}$$ (1)

In the calculation formula, *I*_*m*_(*t*) and *V*_*m*_(*t*) are the value of the current and voltage after normalization. The unit of *I*_*m*_(*t*) is A. The unit of *V*_*m*_(*t*) is V. *I*(*t*) and *V*(*t*) are the current and voltage at the present. The unit of *I*(*t*) is A. The unit of *V*(*t*) is V. *I*_*min*_ and *V*_*min*_ are the minimum values of current and voltage at the present steady-state cycle. The unit of *I*_*min*_ is A. The unit of *V*_*min*_ is V. *I*_*max*_ and *V*_*max*_ are the maximum values of current and voltage at the present steady-state cycle. The unit of *I*_*max*_ is A. The unit of *V*_*max*_ is V. The value of the minimum and maximum is up to the maximum and minimum values of each electrical device data, not fixed.

### Self-incrementing class learning non-intrusive load identification method

**Fig 2:**
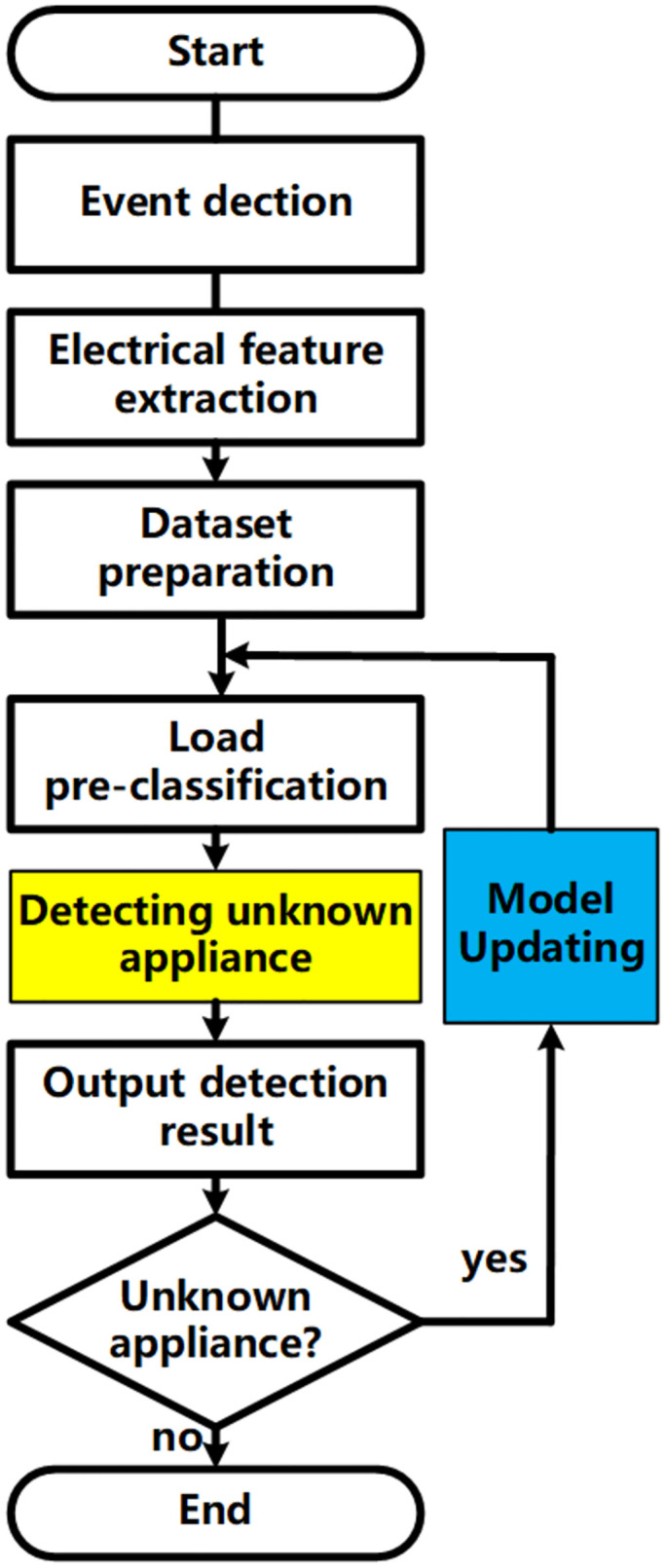
Overall process of self-incrementing class learning for unknown loads.

A non-intrusive load identification method is proposed in this paper for the self-incrementing class learning of unknown loads, based on the V-I trajectory. The complete process of load identification is illustrated in Fig 2. Once data processing and event detection are accomplished, and the binary V-I trajectories dataset is obtained. The chapter primarily focuses on three aspects: load pre-classification modeling, detection of unknown loads, and learning to update the model for unknown loads.

#### Load pre-classification model based on LeNet label acquisition

**Fig 3:**
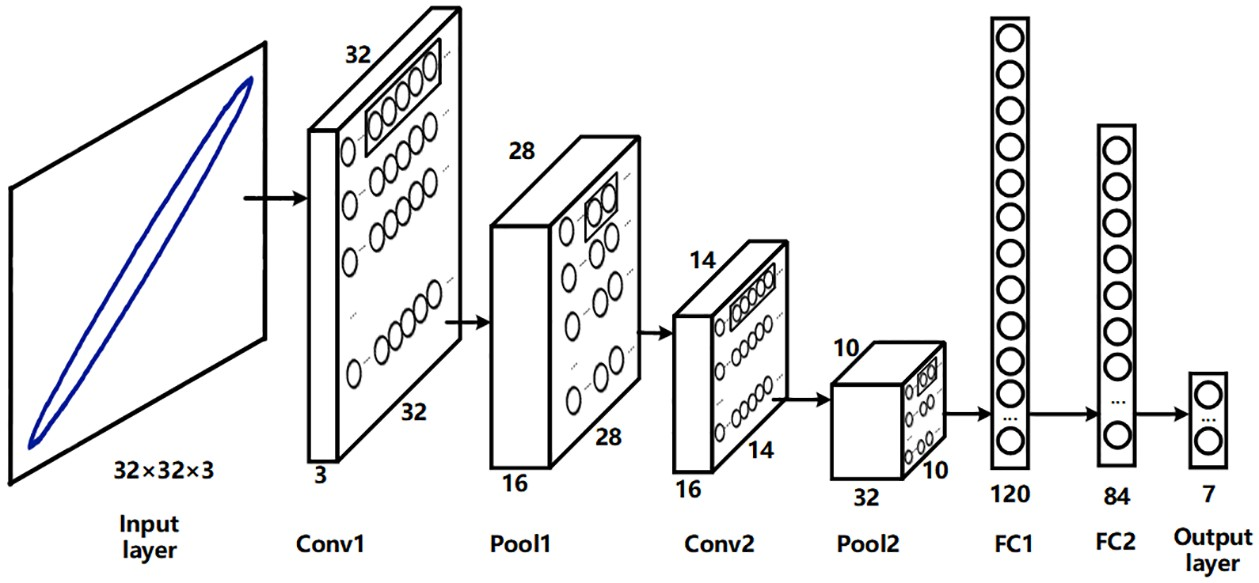
LeNet neural network model.

In recent years, the convolutional neural network (CNN) has gained significant popularity in deep learning and has been widely employed in the field of image processing. Good results have been achieved by CNNs in load classification based on V-I trajectory. Due to the V-I trajectory image containing only one curve and lacking complex background. Consequently, there is no need to opt for a complex deep convolution model. Therefore, in this paper, the classic LeNet [24] model is chosen as the load pre-classification model. The original LeNet model is slightly modified by replacing all sigmoid [25] activation functions with the Rectified Linear Unit [26, 27] (ReLU) activation function. The specific network structure is presented in the Fig 3. The input to the model is a three-channel image with dimensions of 32×32, and the output corresponds to the labels of the 7 known categories of electrical appliances. The detailed parameter settings of the LeNet model can be found in the Table 1.

**Table 1:** Parameter settings of the LeNet model.

Layer Name	Channel	Size	Kernel Size	Stride	Function
Inupt	3	(32×32)	-	-	-
Conv1	16	(28×28)	(5×5)	1	ReLu
Pool1	16	(14×14)	(2×2)	1	Max
Conv2	32	(10×10)	(5×5)	1	ReLu
Pool2	32	(5×5)	(2×2)	1	Max
FC1	-	-	120	-	ReLu
FC2	-	-	84	-	ReLu
Output	-	-	7	-	Softmax

Among the layer names, the first layer of convolution is denoted as Conv1, the first layer of pooling as Pool1, and the first layer of full connection as FC1. The remaining layers follow a similar naming convention. Channel represents the number of channels of each layer feature map. Size represents the size of each layer of feature maps. Kernel size represents the length and width of the convolution kernel. Stride represents the stride size of each convolution movement. The function represents the activation function type of the current layer and the pooling method adopted by the pooling layer. The activation function uses ReLu, and the pooling method adopts max pooling [28, 29]. The number of neurons in the output layer is 7, which represents the number of categories of electrical appliances participating in the training. Additionally, the softmax [30] function is used to map the output to the [0, 1] interval. The maximum probability index is the class for which the load is identified. The calculation of the feature map size for each layer output is given by formula (2).

$$S_{O}=(\frac{I_{S}-F_{S}}{stride})+1$$ (2)

In the formula, *S*_*O*_ is the size of the feature map output by each layer. The unit of *S*_*O*_ is pixel. *I*_*S*_ is the size of the feature map input for each layer. The unit of *I*_*S*_ is pixel. *F*_*S*_ is the size of the convolution kernel of each layer. The unit of *F*_*S*_ is pixel. *stride* is the step size of each layer of convolution kernel movement. The unit of *stride* is pixel. The operation of rounding down is performed after the final calculation is obtained.

#### Unknown load detection method based on combined TNCD method

**Fig 4:**
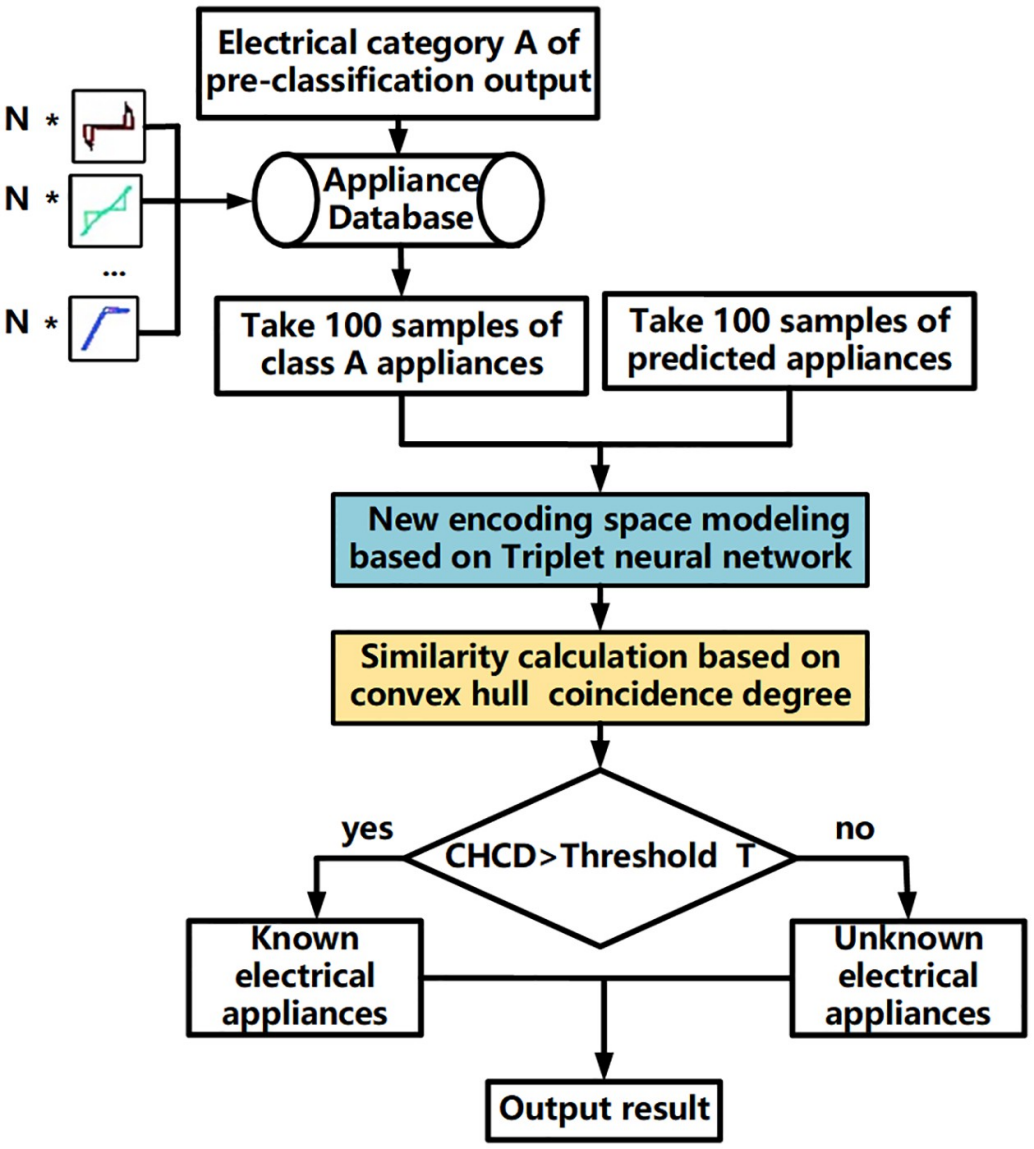
Unknown load detection based on the combined TNCD method.

After load pre-classification of the input load data, the predicted load label is obtained, and it is necessary to ensure that the input load corresponds to the intended load. To address this, a combined method called TNCD (Triplet Neural Network and Convex Hull Coincidence Degree) is proposed for detecting unknown loads. In this method, a new encoding feature space model is generated by a Triplet neural network (blue bar in Fig 4). The similarity calculation method used to distinguish unknown loads is the convex hull coincidence degree (CHCD) (yellow bar in Fig 4). The combined TNCD method, which incorporates both the Triplet neural network and the CHCD, is employed for detecting unknown loads. The complete illustration of the TNCD method for unknown load detection is presented in Fig 4.

#### A.Modeling of new encoding feature spaces based on Triplet neural network

**Fig 5:**
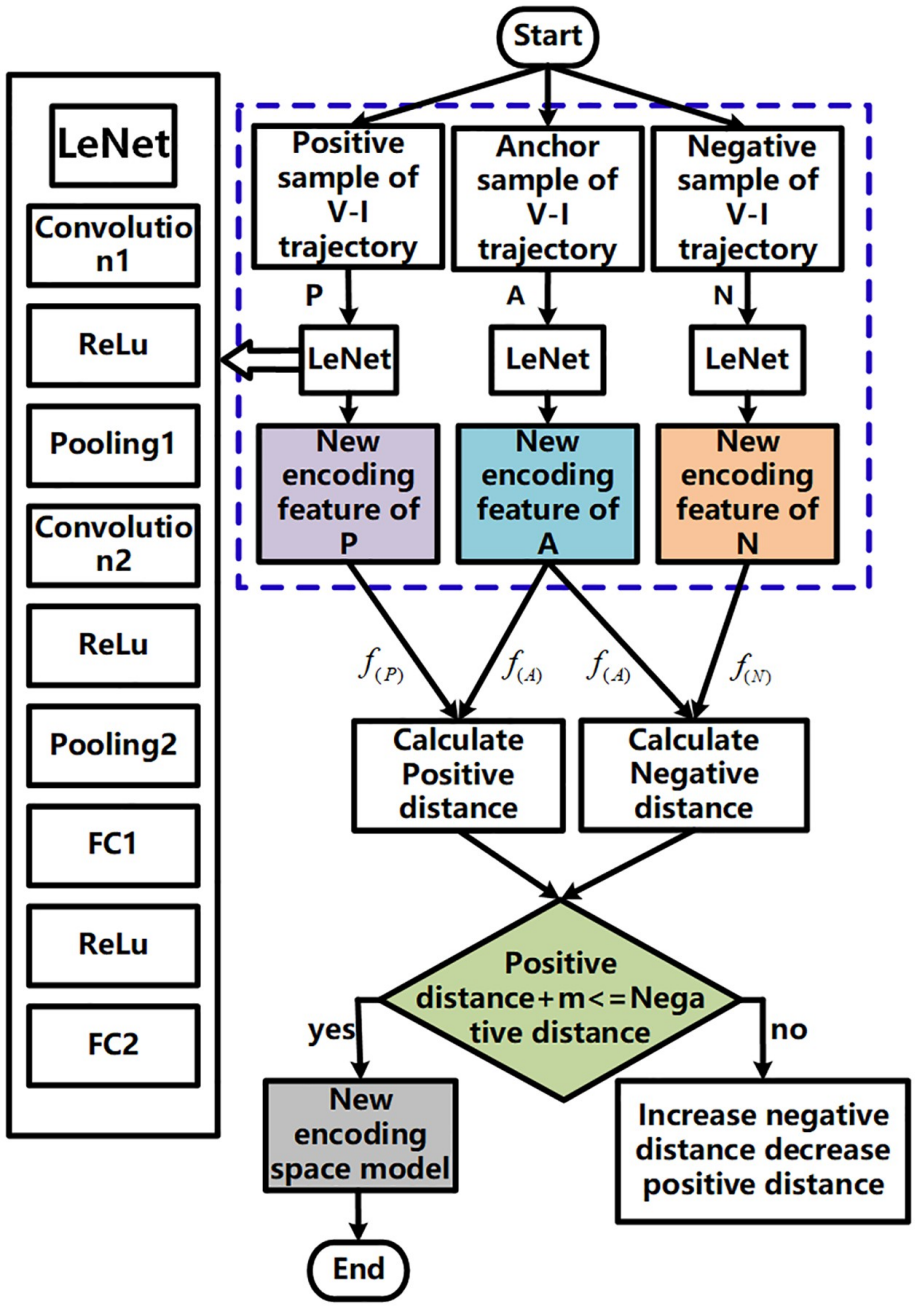
Flow chart for generating a new encoding feature space model based on a Triplet neural network.

The advantage of Triplet [31] neural network clustering, as compared to traditional clustering methods, lies in its ability to automatically determine the number and categories of clusters without pre-setting them. This is achieved through the encoding of input data and similarity calculation. Furthermore, the Triplet neural network can dynamically update the encoding and clustering center, leading to improved accuracy and robustness in clustering. Another strength of the Triplet neural network is its capability to address scenarios where there are fewer categories but a large number of samples within each category, effectively handling imbalanced datasets. In this paper, while the amount of data used for training the network is sufficient, the number of sample data available for identifying unknown loads is relatively small. To address this issue, the Triplet neural network is employed to overcome the challenges associated with small sample data, such as poor category stability and lengthy update times in traditional clustering models. By utilizing the Triplet neural network, the similarity between V-I trajectory image samples is learned, enabling the establishment of a new encoding feature space. Importantly, there is no need to update the Triplet neural network model online to effectively distinguish new categories, thereby achieving real-time performance objectives. The input of the Triplet neural network consists of three V-I trajectory images: an anchor sample, a positive sample from the same category as the anchor sample, and a negative sample from a different category. The output of the Triplet neural network is a vector representing a low-dimensional representation of the anchor samples. The process of establishing a new encoding feature space model using the Triplet network is illustrated in Fig 5.

The architecture of the Triplet neural network is depicted within the blue dotted box. Firstly, the anchor sample (A), positive sample (P), and negative sample (N) are fed into three separate LeNets (as indicated in the solid line box on the left side of Fig 5) to obtain the new encoding features for each sample. To assess the similarity between samples, the positive distance and negative distance are calculated. The positive distance represents the distance between the anchor sample and the positive sample, while the negative distance represents the distance between the anchor sample and the negative sample. These distances are then adjusted using a defined loss function, as computed by the formula (3). The objective of this loss function is to increase the negative distance and decrease the positive distance.

$$L=\{\begin{array}{ll} \sum_{i=1}^{M}||f\left(x_{i}^{A}\right)-f\left(x_{i}^{P}\right)|{|}^{2}-||f\left(x_{i}^{A}\right)-f\left(x_{i}^{N}\right)|{|}^{2}+\alpha & L>0\\ 0 & L\leq 0 \end{array}$$ (3)

In the formula, *M* represents the number of samples used by the Triplet network training appliance. *A*, *P*, and *N* represent anchor, positive, and negative samples, respectively. *α* is the interval parameter. *f*(*x*) is the new encoding features of the sample and the low-dimensional output of the Triplet neural network. Its dimension information is displayed in the formula (4).

$$f\left(x\right)=(f_{x}\left(x\right),f_{y}\left(x\right))$$ (4)

#### B.Unknown load detection based on convex hull coincidence degree

**Fig 6:**
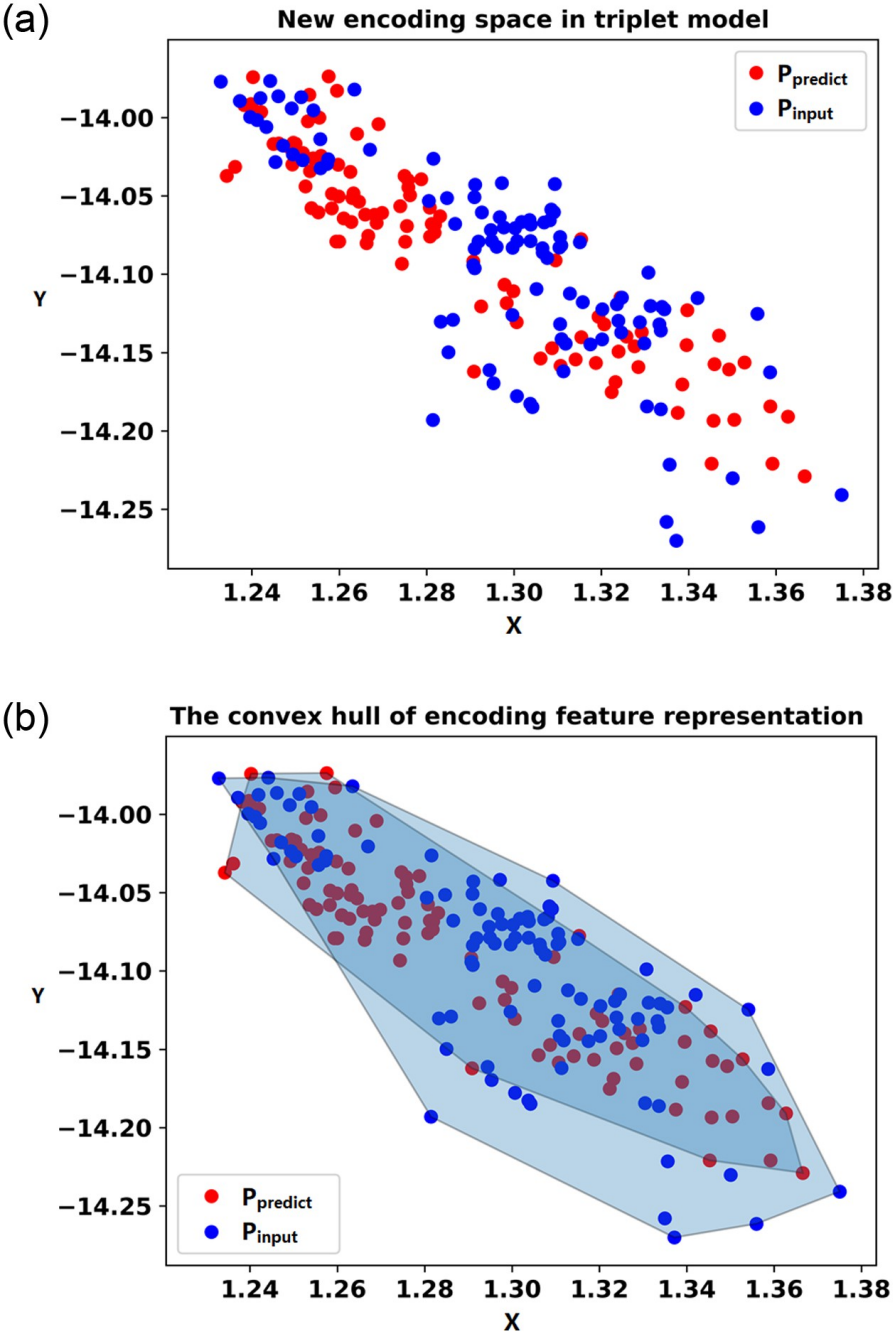
New encoding space representation and the convex hull.

In Fig 6(a), the input load and the load to be detected are indicated by blue points and red points, respectively, on the two-dimensional coordinate axis using the new encoding feature space model. The similarity evaluation method of convex hull coincidence degree is utilized in this paper to predict the load and determine whether it is an unknown load. A convex hull is a convex polygon that encompasses all convex combinations of a given set of points. The specific definition of a convex hull can be found in [32]. The calculation steps for CHCD are briefly described as follows:

1. Identify the leftmost, rightmost, uppermost, and lowermost points from the set of points on the two-dimensional coordinate axis, as well as points on any other boundaries. Connect all these points to form a convex polygon, which is referred to as a convex hull.
2. Calculate the areas of the two convex hulls. In Fig 6(b), the polygon formed by the blue scatter points is referred to as *P*_*input*_, and the polygon formed by the red points is referred to as *P*_*predict*_. Calculate the area *S*_*input*_ and *S*_*predict*_ of *P*_*input*_ and *P*_*predict*_ respectively. Then, calculate the area of the common region between the two polygons.
3. Calculate the convex hull coincidence degree. The calculation of CHCD is shown in the formula (5).
  $$\begin{array}{c} CHCD=\frac{S_{input}⋂S_{predict}}{S_{input}+S_{predict}-\left(S_{input}⋂S_{predict}\right)} \end{array}$$ (5)
  The numerator in the formula represents the coincident region of the convex hulls of the encoding feature representation of the known and unknown loads. The denominator in the formula represents the respective regions of the encoding feature representations of the two loads after removing the repeated regions. Thus, the formula calculates the ratio of the coincident area to the area of the two convex hulls (after removing the coincident part). A higher value indicates a higher degree of coincidence and greater similarity in the encoding feature representation regions of the two loads.
4. To determine whether the input load is unknown, a threshold T is set. If the calculated value of CHCD is greater than the threshold T, it is determined that the input load and the predicted load belong to the same category. Otherwise, if the CHCD value is below the threshold T, it indicates that they do not belong to the same category, suggesting that the input load is unknown.

#### C.Self-incrementing class update method based on parameter freezing

**Fig 7:**
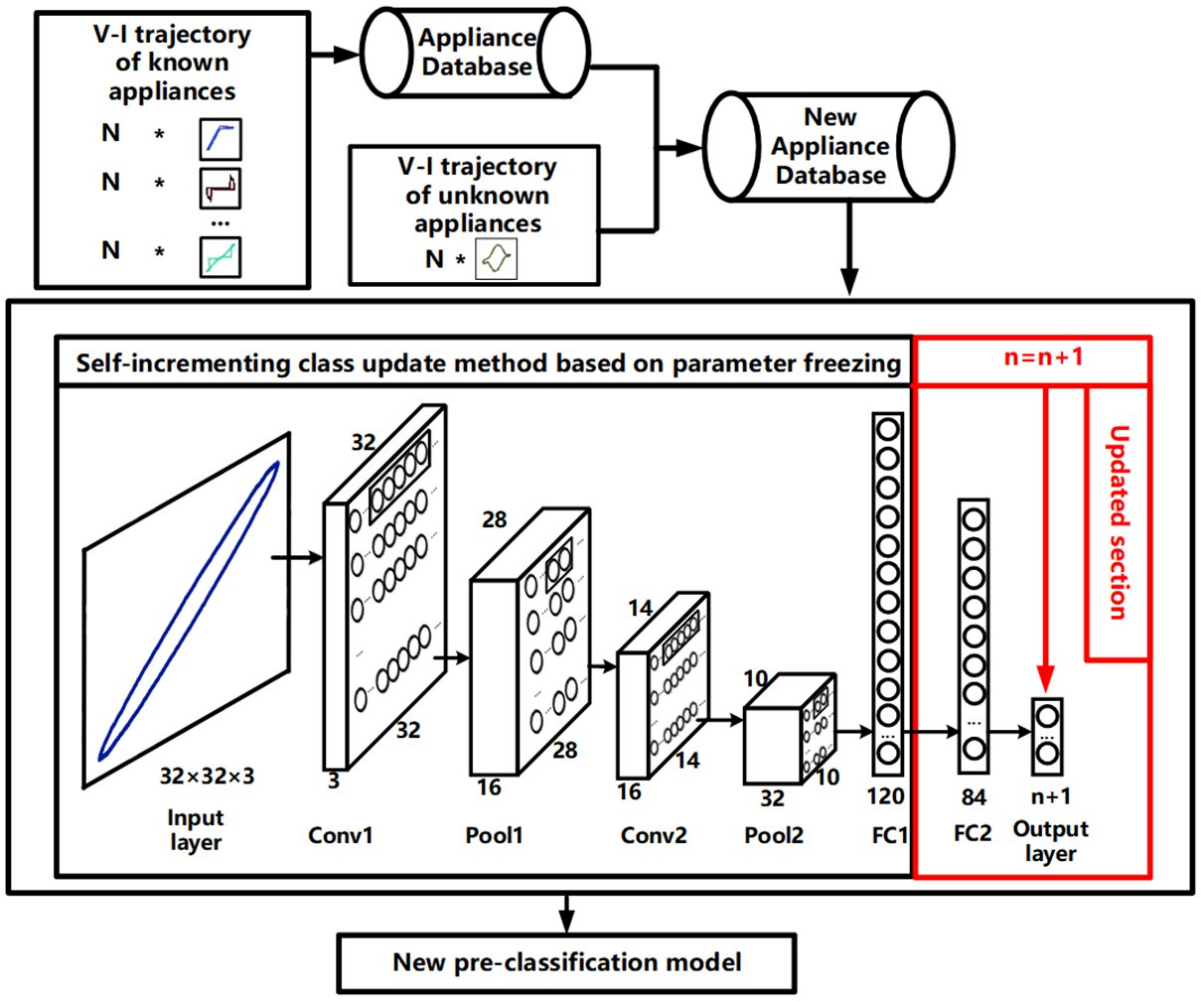
Self-incrementing learning process of the load pre-classification model.

When an unknown load is detected, the load identification model needs to be updated. However, retraining the model can be time-consuming and computationally expensive, resulting in a waste of computing resources. In this paper, a self-incrementing class update model based on parameter freezing is proposed to facilitate the rapid update of the load identification model. The pre-classification LeNet model (see Fig 3) has already undergone training on the structural patterns of the known loading data. Therefore, it is sufficient to freeze all the parameters of the trained LeNet model, except for the parameters of the FC2 layer and the output layer. This allows feature extraction for unknown loads to be performed using a frozen parameter layer. By adopting this approach, the load identification model can be efficiently updated without the need for extensive retraining, thereby reducing the time and computational resources required for the update process. The extracted feature vector is further input into the FC2 layer and the output layer, with an increased number of neurons, to achieve the objective of incremental update. The original pre-classification model has undergone comprehensive training using a large amount of known load data. The network parameters obtained from this training enable effective extraction of load characteristics. Based on this, only a small amount of data is required for training to enhance the model’s understanding of unknown loads. During the training process, similar known load data is added and mixed with unknown loads to form a training database. This training database is used to facilitate the model’s comprehension of the characteristics of unknown loads. The update process of the pre-classification model is detailed in Fig 7. When an unknown load is detected, the load database is updated in real-time. The original database of known loads is merged with both unknown loads and known loads that share similar characteristics, forming a new database. After freezing the parameters, all the data from the new database is input as training data into the pre-classification model. The output feature vector is then input into the updated section of the model, and a new category is added. The number of neurons in the output layer is set to n+1, where n represents the number of load categories in the pre-classification model. The newly added neurons correspond to the number of newly introduced unknown loads. The network is trained to obtain a new pre-classification model, which significantly saves training time and computing resources.

## Results

**Fig 8:**
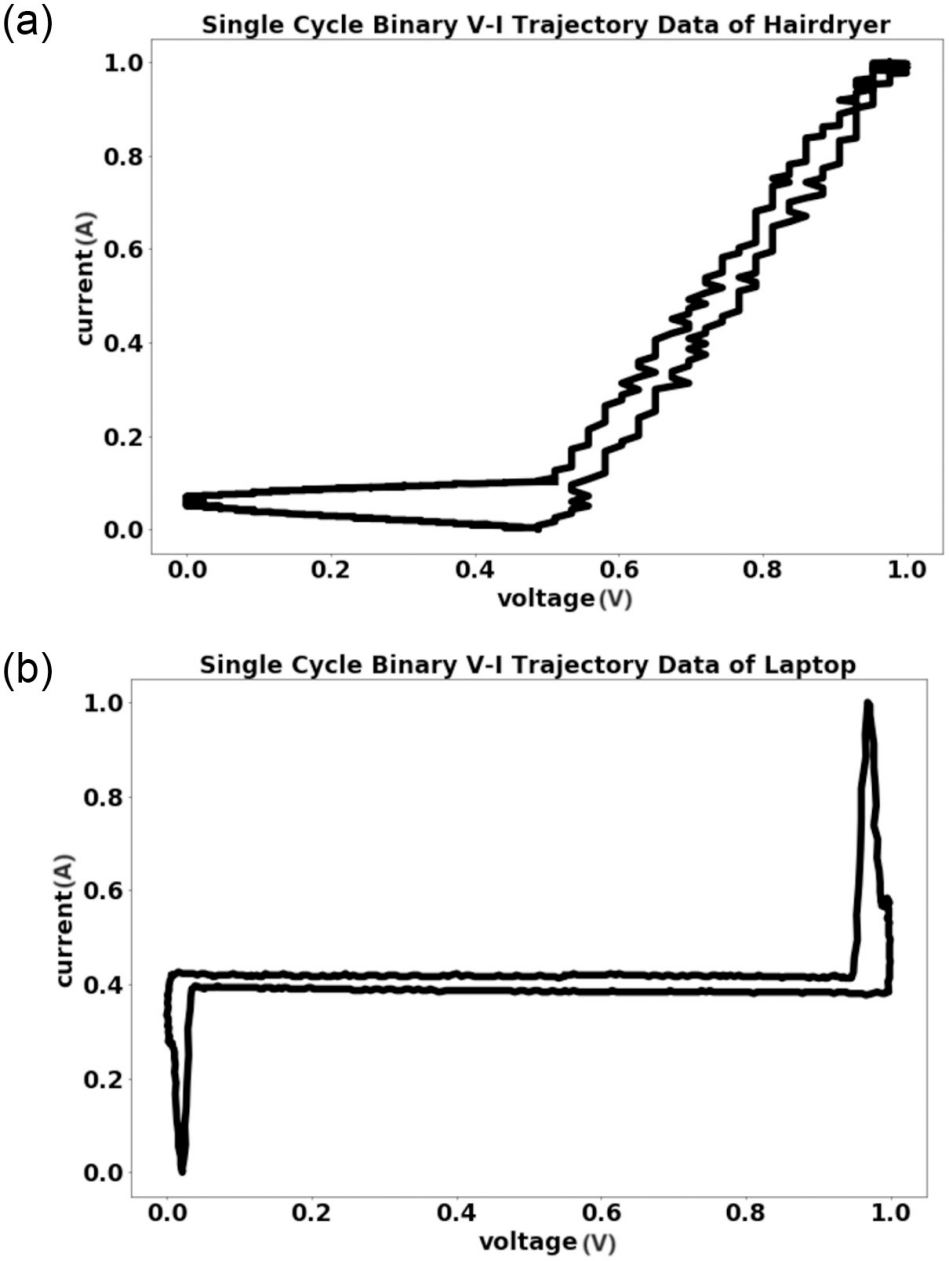
The single cycle binary V-I trajectory pictures of some electrical appliances.

To evaluate the effectiveness of the method proposed in this paper, experiments were conducted from four aspects: binary V-I trajectory discrimination, load pre-classification model reliability, the applicability of the unknown load detection method, and time-saving of the pre-classification model update. The mainstream load public datasets, including PLAID [33], BLUED [34], and REDD [35], were considered. Among these datasets, the PLAID dataset was chosen for this experiment due to its larger data volume, richer data diversity, and higher data quality. Table 2 provides the names of electrical appliances and their corresponding labels. To generate the dataset, 15,000 pictures of binary V-I trajectories were drawn for each type of electrical appliance, with 500 sampling points per period, and a picture was randomly selected from the steady-state period. Known loads were labeled from 0 to 6, while unknown loads were labeled from 7 to 10. The binary V-I trajectory pictures of some electrical appliances are illustrated in Fig 8.

**Table 2:** Load label and name.

0	Air Conditioner
1	Compact Fluorescent Lamp
2	Fan
3	Fridge
4	Hairdryer
5	Heater
6	Incandescent Light Bulb
7	Laptop
8	Microwave
9	Vacuum
10	Washing Machine

### Pre-classification of known loads

**Fig 9:**
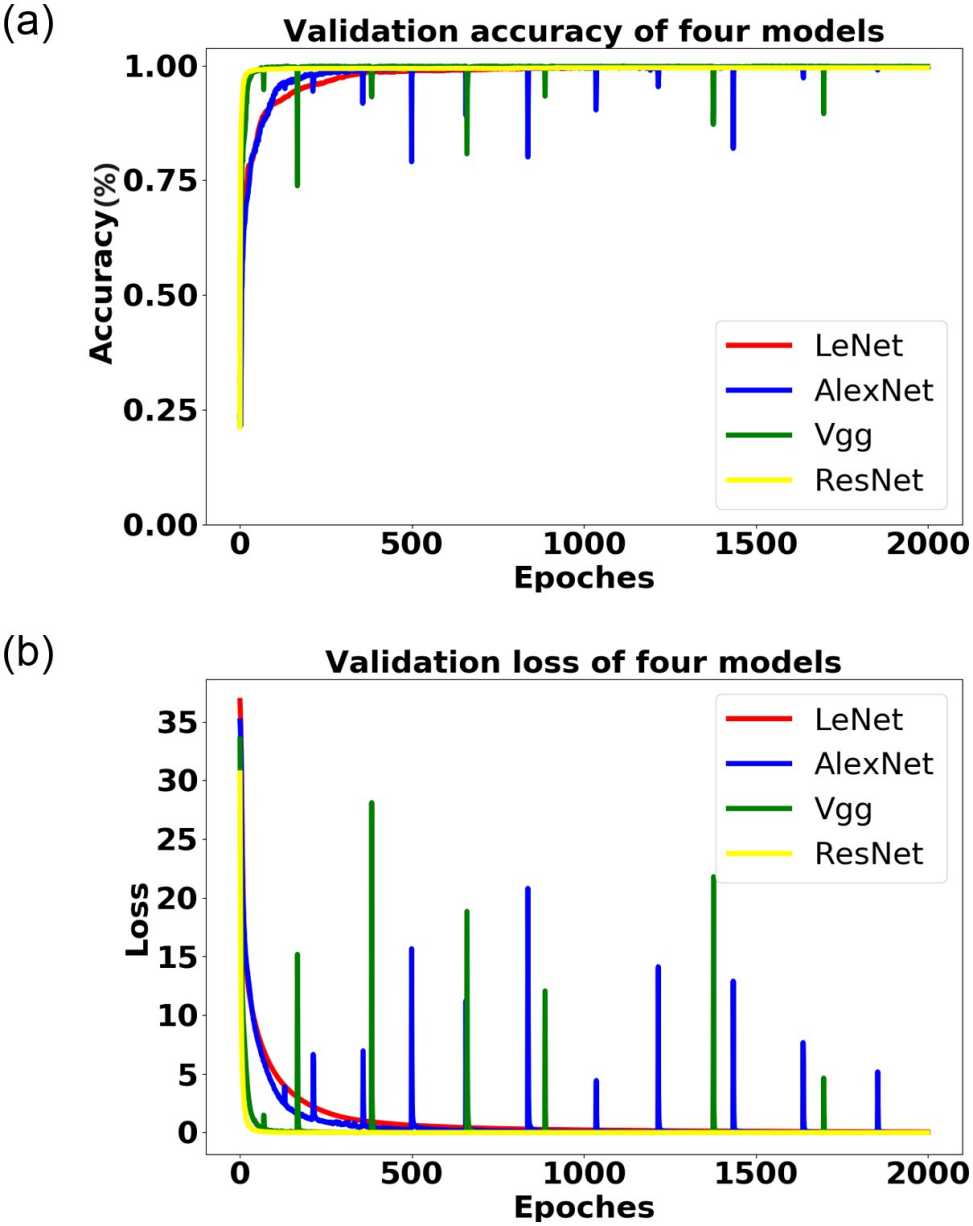
Accuracy and loss comparison of the LeNet, AlexNet, VGG, and ResNet models on the validation set.

The 15,000 samples with known loads were divided into a training set and a validation set in a 7:3 ratio. The training data was then used to train the improved LeNet model proposed in previous chapters, with the parameter settings provided in Table 1. Additionally, three classic convolutional neural networks with different depths, namely Alexnet [36], Vgg16 [37], and Resnet18 [38], compare with LeNet. The same datasets and batch size parameters as described earlier were used for training. Each of these four networks was trained by iterating over the training data 2000 times, resulting in the training of four pre-classification models. The performance of these models was evaluated using the validation set. The detailed result values can be found in Table 3. The table shows that the accuracy of the four models on the validation set is approximately 99%. However, it is worth noting that the LeNet model has a training speed that is 10-20 times faster than the other three models. Analyzing the model loss curve and the accuracy curve on the validation set depicted in Fig 9. The red curve representing the LeNet model exhibits minimal fluctuations. This indicates that the LeNet model is the most stable among the experimental models. Since the V-I trajectory images consist of a single curve without complex backgrounds. The shallow neural network architecture of LeNet is capable of achieving excellent results in load classification based on the V-I trajectory. Consequently, the decision was made to select the LeNet model as the pre-classification model for load identification.

**Table 3:** The result values of the four models.

Network Name	Validation Set Accuracy	Training speed	Final Loss Value	Convergence
LeNet	0.9941	**70item/s**	0.0012	400
Alexnet	0.9953	5.5item/s	0.0007	280
Vgg	0.9965	2.4item/s	0.0006	15
Resnet18	0.9982	6.0item/s	0.0001	10

### Unknown load detection

**Fig 10:**
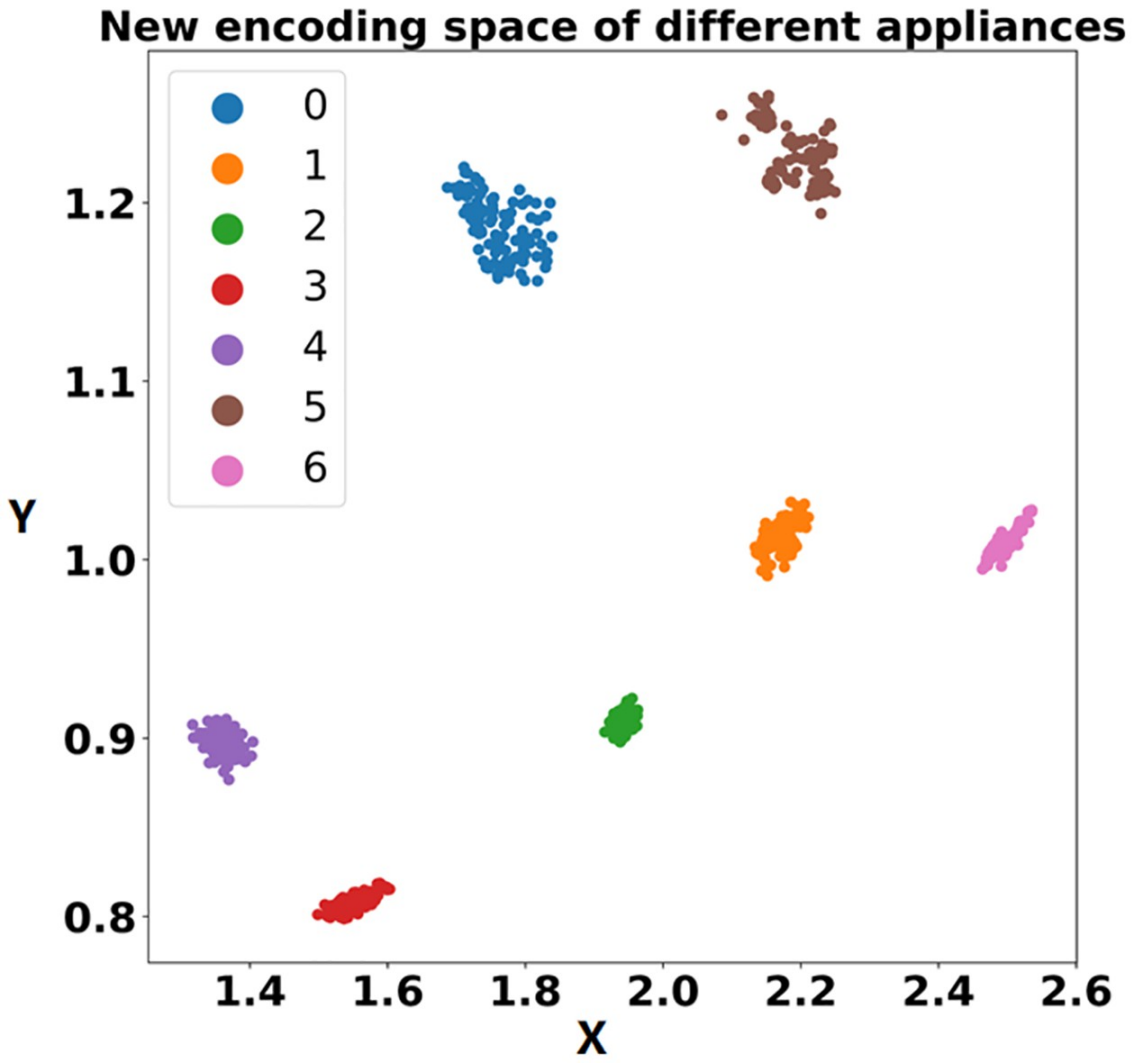
New encoding space representation of data from 7 known loads.

The detection methods for unknown loads were examined based on the accurate identification of known loads by pre-classification models. Following the process of establishing a new encoding feature space based on Triplet (see Fig 5), a new encoding feature space was established for the 7 known loads. The training set was split according to a ratio of 7 to 3 for the training set and validation set. A batch size of 64 was set, the Triplet neural network learning rate was set to 0.01, and the margin was set to 1.0. The model parameters were optimized using the formula (3), which calculates the Triplet loss. After 1000 rounds of iterations, the new encoding feature space was finally obtained, as depicted in Fig 10.

**Fig 11:**
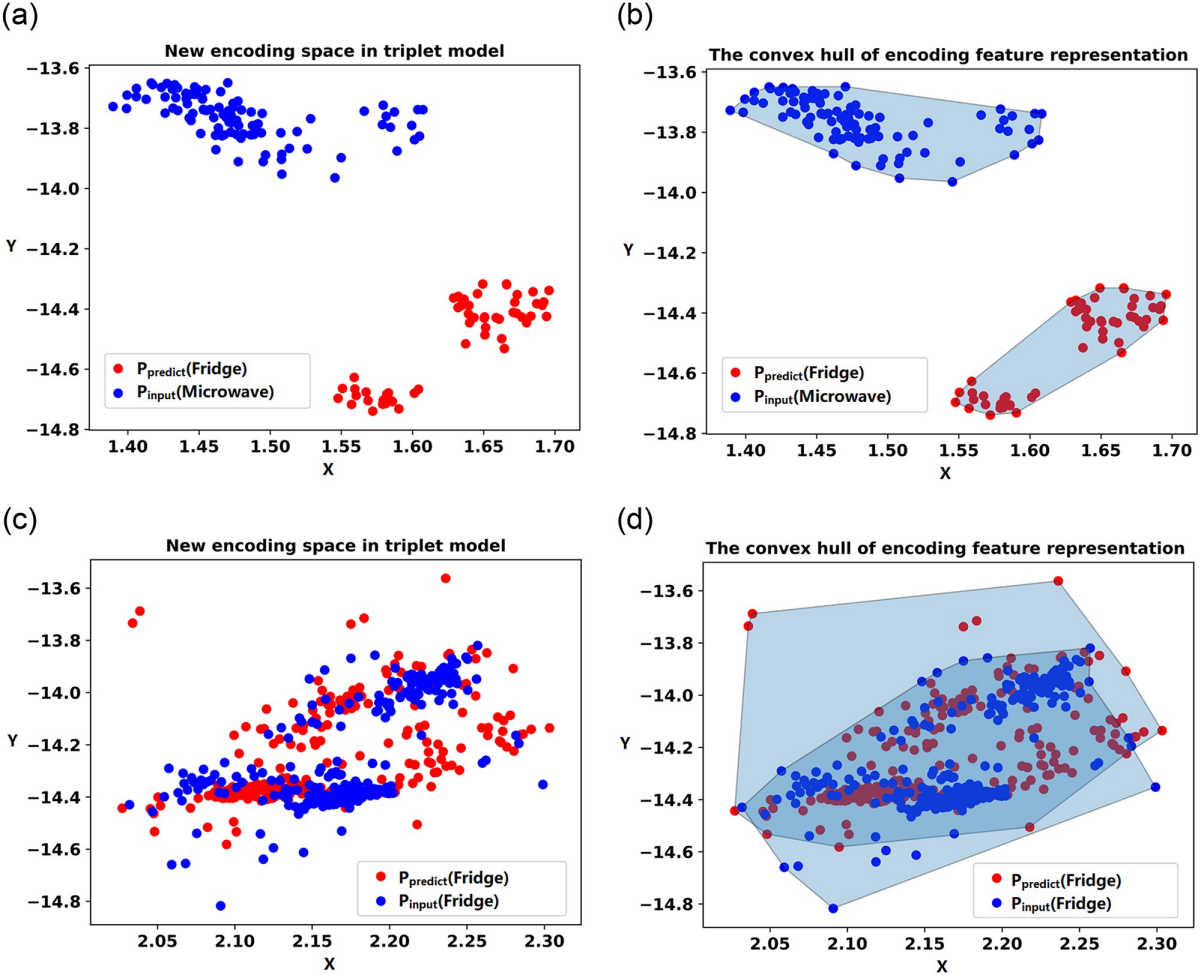
Unknown load detection process for unknown loads and known loads.

Each load sample is represented by different colored scatter points in the process of mapping to the two-dimensional coordinate axis. It can be observed from the figure that the new encoding model clearly distinguishes each load and maps them to different regions in the two-dimensional space. After the construction of the new encoding feature space model, the unknown load is detected using the combined TNCD method as shown in Fig 4. The detection process of unknown loads is illustrated using the example of the Microwave device with label 7. Firstly, the pre-classification model trained in previous chapters is utilized to input the Microwave sample, obtaining the label of the known load fridge that exhibits the most similar characteristics. Following the process depicted in Fig 4, a selection of 100 fridge samples and 100 Microwave samples are made. These 200 samples are then input into the new encoding feature space model to derive their respective new coded feature representations. In the Fig 11(a), the blue point signifies the input load, representing the new encoding feature space of the Microwave. The red point represents the predicted load, representing the new encoding feature space of the fridge. Both the blue and red points reside within their respective regions, displaying a noticeable distance between them. Subsequently, a convex hull is constructed based on the new encoding feature space representations of the two loads, as depicted in Fig 11(b).

**Fig 12:**
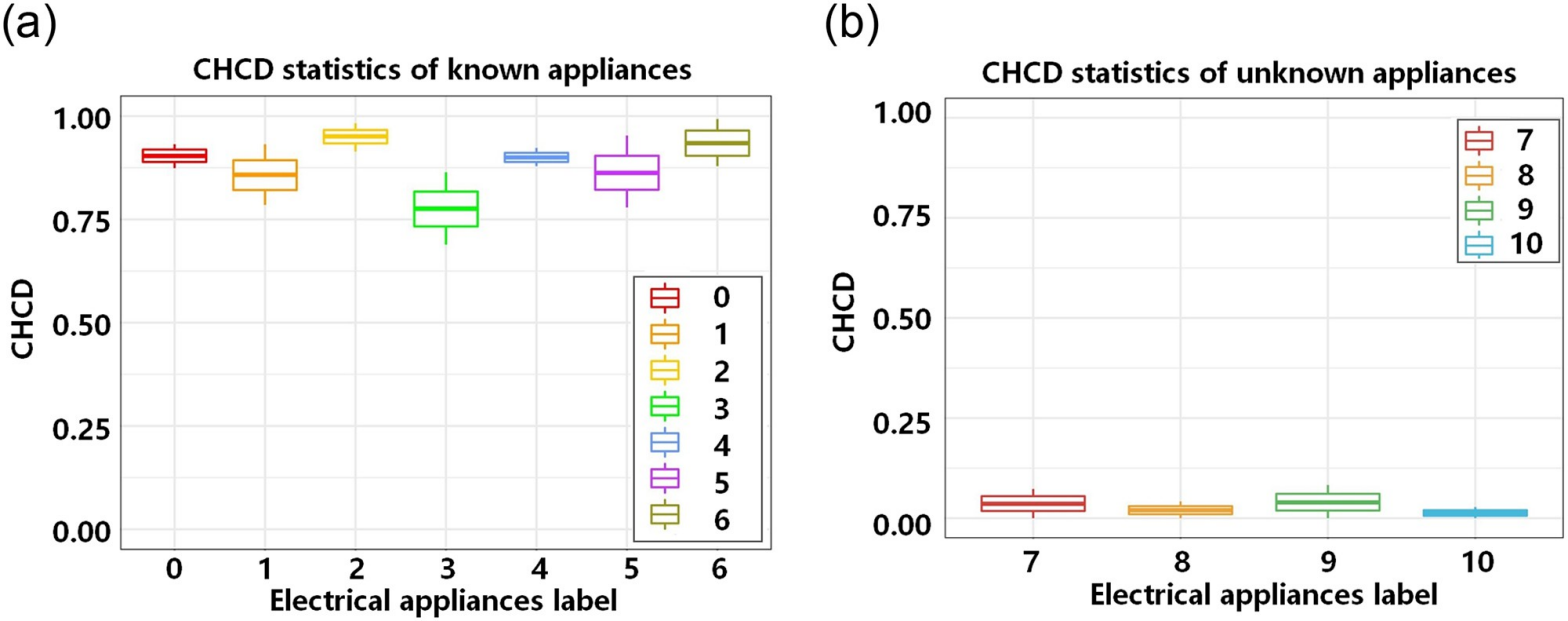
CHCD statistical box plot of load data feature space representation.

Finally, the CHCD is computed for the two convex hulls, utilizing CHCD as the criterion to assess the similarity between the loads. In the figure, the calculated CHCD for the two convex hulls is determined to be zero, indicating that the two loads are dissimilar and not the same load. Subsequently, fridge samples are again selected from the known load database, and the aforementioned detection process is repeated. The pre-classification model outputs a label of the fridge, their respective new coded feature representations are shown in Fig 11(c), and their respective convex hulls are shown in Fig 11(d). The calculated value of the convex hull coincidence degree is 0.873, indicating a high similarity between the two loads and confirming that they belong to the same load type. To further evaluate the detection process, 300 experiments are conducted on each of the 11 load types, resulting in two box plots. In Fig 12(a), it can be observed that the CHCD values for known loads are consistently above 0.65. Similarly, Fig 12(b) demonstrates that the CHCD values for unknown loads are all below 0.2. These findings indicate that setting the CHCD threshold to 0.65 enables accurate differentiation between known and unknown loads.

### Self-incrementing class update of pre-classification model

**Fig 13:**
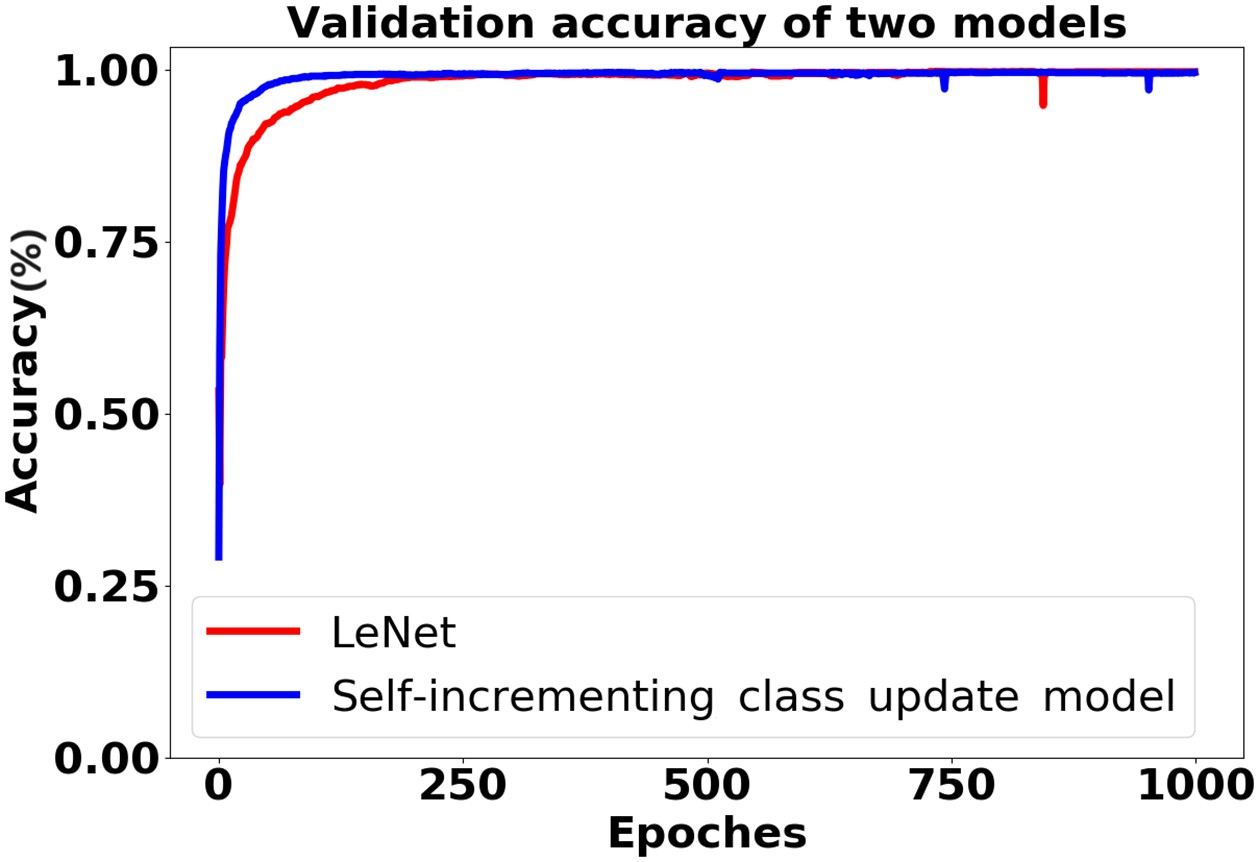
Comparison of accuracy values between the self-incrementing learning model and the LeNet model.

**Fig 14:**
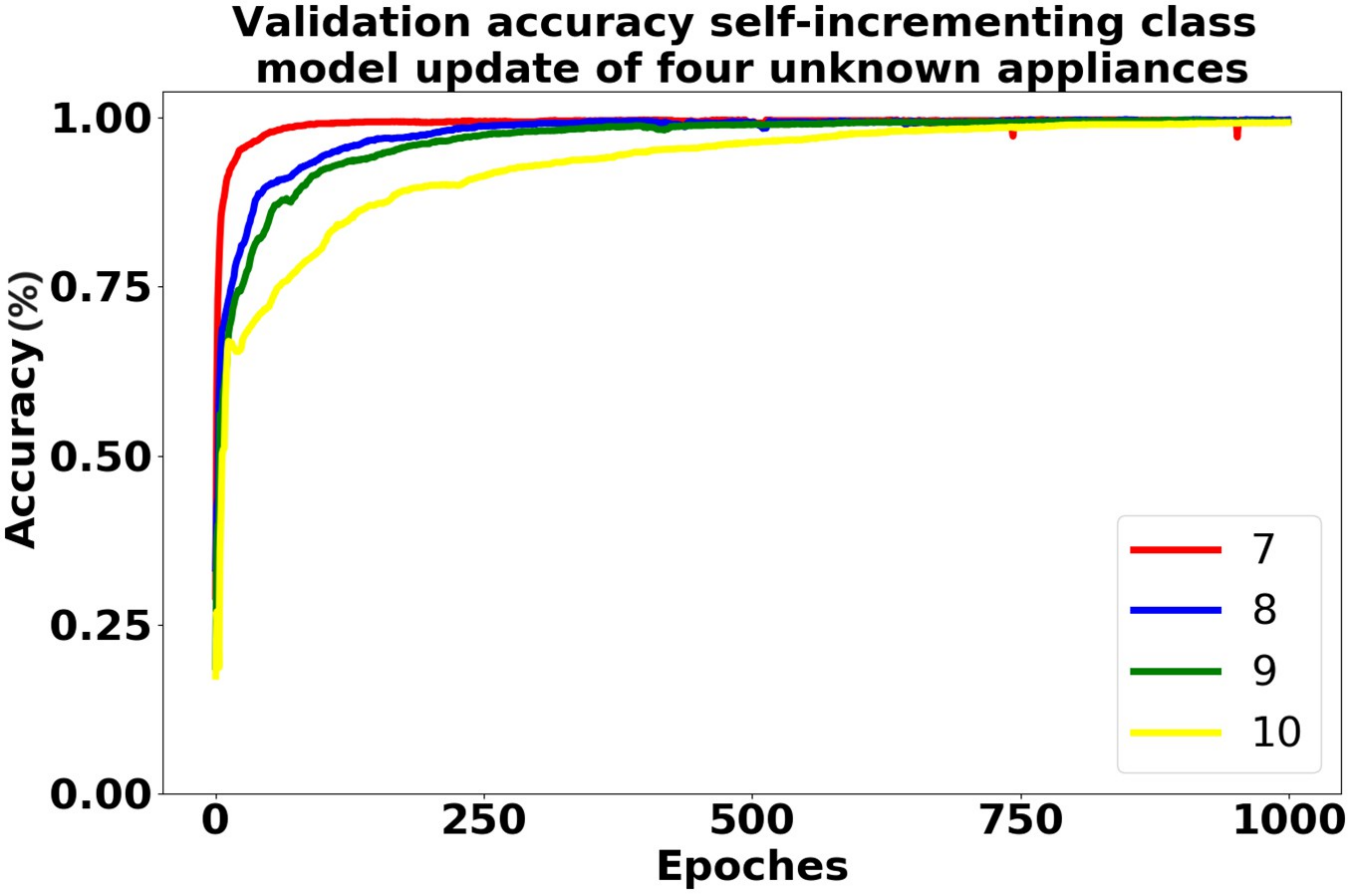
Accuracy comparison of self-incrementing learning model learning four unknown loads in order.

After the unknown load is detected, the pre-classification model is updated using the self-incrementing class method, following the process depicted in Fig 7. The training data is split into a train set and a validation set with a ratio of 7 to 3, and the iteration is performed 1000 times. The training results of regular training and self-incrementing class update with the addition of Microwave based on the 7 known loads are presented in Table 4. Through analysis, it is discovered that the self-incrementing class update requires less training time, and the accuracy of the two methods is comparable. This achieves the objective of quickly updating the model without compromising accuracy. The training accuracy of both models on the validation set is illustrated in Fig 13. Continuing the experiment after incorporating Microwave, the remaining three unknown loads are sequentially trained using the self-incrementing class update method. Table 1 provides a detailed overview of the various parameters after each load training is completed. The training accuracy on the validation set for the self-incrementing learning model with the four types of electrical appliances is presented in Fig 14. All accuracies exceed 90%, and the training speed is notably fast. This fully validates the efficacy of this method in achieving rapid self-incrementing class updates on the pre-classification model while maintaining a high accuracy rate.

**Table 4:** The result values of the two models.

Method	Iteration					Time
-	10	50	100	500	1000	-
LeNet	**0.2254**	0.6934	0.8414	0.9783	0.9865	551
Self-incrementing class update model	0.1764	**0.9304**	**0.97**	**0.9908**	**0.9923**	**73**

## Discussion

Combining each experimental part of this paper, discussing the experimental results: The method proposed in this paper utilizes the low-dimensional mapping ability of the Triplet neural network and employs self-incrementing class learning based on model parameter freezing. Our known load pre-classification model based on the LeNet neural network exhibits high accuracy. In the detection of unknown equipment, the combined TNCD method addresses the instability of traditional clustering methods and effectively identifies unknown loads. Through the utilization of the self-incrementing learning model based on network parameter freezing, the pre-classification model can be rapidly updated following the identification of unknown loads. The experiments conducted on 11 different load types achieved an average accuracy rate of 99.23%. So, the method proposed in this paper exhibits higher accuracy compared to the previous methods. Additionally, the proposed method expands the functionality of the NILM system by enabling the identification and modeling of unknown loads. However, it should be noted that the method proposed in this paper also has limitations, such as the consumption of additional computing resources due to the combination of different algorithms.

## Conclusion

In this paper, a method is proposed to address the challenge of identifying unknown loads by combining TNCD. The Triplet neural network is employed to establish a new encoding feature space model. The benefit of the Triplet neural network is to map similar samples to adjacent locations in the embedding space while mapping dissimilar samples to distant locations. Then combine the convex hull coincidence degree to detect the unknown loads. Finally, transfer learning is applied to update the pre-classification model through self-incrementing classes. Transfer learning can improve the generalization ability of a model, making it perform better on unknown data. The knowledge originally trained helps the model better understand and capture the common characteristics of new data. These advantages can save a lot of time during the training process and quickly update the model. The performance of the proposed method is validated using the publicly available PLAID dataset, demonstrating high accuracy in identifying unknown loads.
